# Subjective Outcome Evaluation Based on Secondary Data Analyses: The Project P.A.T.H.S. in Hong Kong

**DOI:** 10.1100/tsw.2010.4

**Published:** 2010-02-12

**Authors:** Daniel T.L. Shek, Rachel C.F. Sun

**Affiliations:** ^1^ Department of Applied Social Sciences, The Hong Kong Polytechnic University, Hong Kong; ^2^ Department of Sociology, East China Normal University, Shanghai, China; ^3^ Kiang Wu Nursing College of Macau, Macao; ^4^ Department of Social Work, The Chinese University of Hong Kong, Hong Kong

**Keywords:** positive youth development program, secondary data analyses, subjective outcome evaluation

## Abstract

The intent of this study was to evaluate the program effectiveness of the Project P.A.T.H.S. (Positive Adolescent Training through Holistic Social Programmes) (Secondary 1 Curriculum) by analyzing 207 school-based program reports, in which program implementers were invited to write down five conclusions based on an integration of the subjective outcome evaluation data collected from the program participants and program implementers. Secondary data analyses were conducted and 1,855 meaningful units were extracted from 1,035 “aggregated” conclusions. Among them, about 27 and 18% were related to perceptions of the program and implementers, respectively, and most of them were positive in nature. About one-third was related to perceived effectiveness of the program, and most of them referred to enhancement of students' development in societal, familial, interpersonal, and personal aspects. However, difficulties encountered during program implementation (3.34%) and recommendations for improvement (18.11%) were also reported. The present study replicated the findings reported in previous studies and suggests that the Tier 1 Program of the Project P.A.T.H.S. is beneficial to the development of the program participants.

### INTRODUCTION

To promote holistic development among early adolescents in Hong Kong, The Hong Kong Jockey Club Charities Trust launched a project entitled "P.A.T.H.S. to Adulthood: A Jockey Club Youth Enhancement Scheme", with "P.A.T.H.S." standing for Positive Adolescent Training through Holistic Social Programmes[1,2]. In the project, there are two tiers of programs. The Tier 1 Program is a universal, positive youth development program for Secondary 1 to 3 students, whereas the Tier 2 Program is a selective program for those who have greater psychosocial needs. The present study focused on evaluating the effectiveness of the Tier 1 Program, which was designed by the research team with reference to the positive youth development constructs proposed by Catalano et al.[3], research findings, emerging and local youth issues, and Chinese culture. A set of curriculum manuals was developed and distributed to the participating schools, and adequate training was provided for teachers and social workers who implemented the program.

The effectiveness of the Tier 1 Program in promoting holistic and positive development among early adolescents has been evidenced by various evaluation findings generated from different methods and data sources, including longitudinal objective outcome evaluation[4,5], subjective outcome evaluation collected from the program participants and implementers[6,7,8,9,10], qualitative evaluation based on focus group interviews with students and instructors[11,12,13,14], analyses of students' weekly diaries[15] and case studies[16,17], process evaluation based on systematic classroom observation[18,19], and interim evaluation collected from the views of program implementers[20,21]. All these findings consistently showed that both program participants and implementers had positive perceptions of the program and regarded the program as helpful to the students' overall development, and the participants displayed positive changes after joining the program.

Among these evaluation studies, most of them were conducted by the research team members, except subjective outcome evaluation. The purposes of subjective outcome evaluation were to examine the views of program participants and implementers on program effectiveness and their satisfaction levels of the program. While the subjective outcome evaluation forms and the procedures of data collection and analyses were designed by the research team, the program implementers (i.e., teachers or social workers) were responsible for data collection and report writing. Such an arrangement, on one hand, was based on the premise that program implementers were the most suitable people to evaluate the program implementation in their own ecological contexts and, on the other hand, was in anticipation of promoting practice evaluation in the field. After completion of the Tier 1 Program, program implementers of each participating school were required to collect completed subjective outcome evaluation forms from the program participants (Form A) and program implementers (Form B). Based on an integration of the evaluation findings using Form A and Form B, they were required to draw five conclusions as part of the school-based evaluation report. An evaluation manual was provided to the program implementers as a self-help tool, and adequate training was provided in order to equip them with the necessary skills and attitudes to conduct subjective outcome evaluation of the program.

Practically, there are several arguments against the use of evaluation data collected by the program implementers[10]. First, it is doubtful whether program implementers would have the required expertise to conduct evaluations. Second, if the program implementers have to perform as evaluators as well, it is argued that there may be role conflict and confusion. Third, it is argued that the credibility of the data collected will be affected if the program implementers could not guard against their biases in data collection and analysis. For instance, the implementers may boost the program effectiveness if they have to be accountable for their delivered service or even have to seek their job security (i.e., rice-bowl argument). Also, since program implementers have invested time and effort in order to execute the program, it is difficult for them to evaluate the program in a negative manner (i.e., cognitive dissonance argument). On the other hand, if the program implementers are not totally willing to implement the program, they may consciously or unconsciously evaluate the program in an unfavorable manner (i.e., revenge argument).

Nevertheless, there are several counterarguments that support the involvement of program implementers in the evaluation process. The first and most obvious is that some professionals, such as teachers and social workers in Hong Kong, are trained to conduct evaluation research. In fact, there has been a movement to treat teachers or instructors as "internal evaluators" because they are in the most eligible position to document their own practice and promote innovations in pedagogy[22,23]. Second, professionals are usually expected to carry out program implementation as well as program evaluation, since evaluation is part of practice. Hence, role conflict is basically not a problem if trained teachers and social workers have to be program evaluators. Third, reflective practice is strongly emphasized in these human-service professionals to tackle one's biases and assumptions, and thus sincerity and integrity in evaluating one's performance and program delivery are upheld.

Finally, as the utilization-focused evaluation paradigm advocates the importance of involving different stakeholders in the evaluation process[24], an increasing number of program evaluation studies have gathered not only the views of the beneficiaries (such as students and their parents), but also the opinions of the program implementers, coordinators, administrators, and professionals[25,26,27]. Since it is of paramount importance to gather various "expert" views in order to paint a complete picture of the program effectiveness, collecting the views of program implementers as well as engaging program implementers as evaluators is legitimate. Brandon[28] also echoed the importance of engaging program implementers as one of the stakeholders in program evaluation by arguing that program implementers are more familiar with the program arrangement and operation, as well as students' capability and the teaching context, and thus are in an "expert" position to evaluate the program. Furthermore, it is argued that program implementers can be engaged as program evaluators who are responsible for program assessment and report writing simultaneously because they have the ability to translate evaluation findings into meaningful conclusions in which their proficient views would be useful for program improvement and advancement in the future[29].

By utilizing and integrating the five conclusions drawn in the school-based evaluation reports prepared by the program implementers based on the views of both program participants (Form A) and program implementers (Form B), the present study conducted secondary data analyses to evaluate the effectiveness of the Project P.A.T.H.S. According to Royse[30], secondary data analysis "involves analysis of an existing data set that results in knowledge, interpretations, and conclusions beyond those stated in the original study" (p. 201), and it is a kind of unobtrusive research method, which does not need to have direct interaction with the subjects. Unlike primary research or data analysis, which starts from formulating research questions to designing appropriate methods to collect suitable data, secondary data analysis is usually conducted either by beginning with research questions to searching for a suitable database, or by choosing a database prior to formulating research questions that can be answered by the selected database[31,32]. Obviously, the present study adopted the latter strategy of secondary data analysis. The significance of conducting secondary data analyses was highlighted by Kiecolt and Nathan[33], "our increased familiarity with and use of preexisting data encourage social scientific progress" (p.12).

When performing secondary data analyses, the present study addressed the following questions (why, what, who, when, where, and how - "W5H1" questions) raised by several researchers[31,32]. First of all, "*Why* conduct the study?" The research goal of the present study was to evaluate the effectiveness of the Project P.A.T.H.S. Second, "*What* data were collected and what were they intended to measure?" This study clearly specified that the kind of data to be investigated was qualitative data, which were five conclusions based on an integration of the subjective outcome evaluation findings. With reference to the previous study[10], the aspects of investigation were specified to be the instructors' and students' views of the program, views towards the instructors, perceived program effectiveness, difficulties encountered, and recommendations for program implementation. Third, "*Who* were responsible for data collection and what were their qualifications?" The data were collected by the program implementers (either teachers or social workers) of each school who had received evaluation training in their professional schooling and the instructor training workshops provided by the project prior to program implementation. Fourth, "*When* and *Where* was the information collected?" The data were collected after completion of the Tier 1 Program in each school, whereas the evaluation reports were submitted at term end (i.e., end of summer break in late August 2007). Last, "*How* was the information organized?" Based on an integration of the evaluation findings using Form A and Form B, the program implementers of each school were required to draw five conclusions regarding the program effectiveness in the evaluation report. After receiving the evaluation reports, the research team aggregated the findings and conducted secondary data analyses.

In short, the present study was intended to examine the effectiveness of the Tier 1 Program of the Project P.A.T.H.S. based on the secondary data analyses of conclusions drawn by the program implementers. Although a similar secondary data analysis was conducted[10], that study was based on the analyses of 52 schools' evaluation reports in the Experimental Implementation Phase. To examine the replicability of the findings, secondary data analyses based on 207 school evaluation reports collected in the Full Implementation Phase are reported in this study.

## METHODS

### Dataset for Secondary Data Analyses

In the 2006/07 school year, 207 schools joined the Secondary 1 Curriculum of the Project P.A.T.H.S. in the Full Implementation Phase. After completion of the Tier 1 Program, a total of 33,693 students (mean = 162.77 students per school, range: 15–265) and 1,250 workers (mean = 2.13 social workers per school, range: 0–9; mean = 5.47 teachers per school, range = 0–14) were invited to respond to the Subjective Outcome Evaluation Forms, Form A and Form B, respectively, as designed by Shek and Siu[34]. "Passive" informed consent was obtained from the respondents, in which the purpose of the evaluation was mentioned and the confidentiality of the data collected was emphasized. Adequate time was provided for the respondents to complete the questionnaire in a self-administration format and in an anonymous manner. The data were collected by the program implementers who had received adequate training in data collection and report writing prior to program implementation.

There are several parts in Form A: (a) participants' perceptions of the program, such as program objectives, design, classroom atmosphere, interaction among the students, and the respondents' participation during class (10 items); (b) participants' perceptions of the instructors, such as preparation, professional attitude, involvement, and interaction with the students (10 items); (c) participants' perception of the effectiveness of the program, such as promotion of different psychosocial competencies, resilience, and overall personal development (16 items); (d) the extent to which the participants would recommend the program to other people with similar needs (one item); (e) the extent to which the participants would join similar programs in future (one item); (f) overall satisfaction with the program (one item); (g) things that the participants learned from the program (open-ended question); (h) things that the participants appreciated most (open-ended question); (i) opinion about the instructors (open-ended question); and (j) areas that require improvement (open-ended question).

Similar to Form A, Form B includes the evaluation of (a) program implementers' perceptions of the program, such as program objectives, design, classroom atmosphere, interaction among the students, and the students' participation during class (10 items); (b) program implementers' perceptions of their own practice, including their understanding of the course, teaching skills, professional attitude, involvement, and interaction with the students (10 items); (c) program implementers' perceptions of the effectiveness of the program, such as promotion of different psychosocial competencies, resilience, and overall personal development of the students (16 items); (d) the extent to which the workers would recommend the program to other students with similar needs (one item); (e) the extent to which the workers would teach similar programs in future (one item); (f) overall satisfaction with the program (one item); (g) things that the workers obtained from the program (open-ended question); (h) things that the workers appreciated most (open-ended question); (i) difficulties encountered (open-ended question); and (j) areas that require improvement (open-ended question).

Based on the evaluation data collected in each school, the program implementer in each school was required to complete a Tier 1 Program evaluation report where both quantitative and qualitative findings based on Form A and Form B were summarized and described. In the last section of the report, the program implementer was requested to write down five conclusions regarding the program and its effectiveness. The involvement of the workers in writing the conclusions is consistent with the thesis that program implementers can give a more comprehensive picture about the program quality and benefits to students. Thus, they are proficient in accounting program effectiveness with reference to various aspects, and providing recommendations for improving program arrangement and delivery in the real teaching context[28,29].

### Data Analyses

The data generated from the five conclusions were analyzed using general qualitative analyses techniques[35] by two research assistants, of which one has a Bachelor's Degree in Psychology and another has a Bachelor's Degree in Social Work. The final coding and categorization were further cross-checked by another research colleague with a Master's Degree in Social Work. All the research staff received sufficient training in both quantitative and qualitative analyses. There were three steps in the data analysis process. First, raw codes were developed for words, phrases, and/or sentences that formed meaningful units in each conclusion at the raw response level. Second, the codes were further combined to reflect higher-order attributes at the category of codes level. For example, the response of "satisfied with the program" at the raw response level could be subsumed under the category of "satisfaction level", which could be further subsumed under the broad theme of "views on the program" (see Table 1).

As program developers, the authors might have the preoccupation that the implemented program was good and it was beneficial to the students. Thus, several safeguards against the subtle influence of such ideological biases and preoccupations were included in the present study. First, the authors were conscious of the existence of ideological preoccupations and carried out the analyses in a disciplined manner. Second, both intra- and inter-rater reliability on the coding were calculated in order to minimize the possible biases involved. For intrarater reliability, each of the two research assistants primarily responsible for coding coded 20 randomly selected responses without looking at the original codes given after checking by the authors. For inter-rater reliability, another two trained research assistants (both of them have a Master's Degree) who had not been involved in the data analyses coded the same 20 randomly selected responses without knowing the original codes given at the end of the scoring process after checking by the authors.

Following the principles of qualitative analyses proposed by Shek et al.[36], the following attributes of the study regarding data collection and analyses were highlighted. First, a general qualitative orientation was adopted. Second, the sources of data (e.g., number of participants) for analyses were described. Third, the issues of biases and ideological preoccupation were addressed. Fourth, inter- and intrarater reliability information was presented. Fifth, the categorized data were kept by a systematic filing system in order to ensure that the findings are auditable. Finally, possible explanations, including alternative explanations, are considered.

### RESULTS

Based on the 1,035 conclusions in the 207 evaluation reports, 1,855 meaningful units were extracted. Based on the previous study[10], these raw responses were further categorized into several categories, of which 26.79% related to views on the program (Table 1), 18.01% related to views on the program implementers (Table 2), 33.75% related to perceived general and specific effectiveness of the program (Table 3), 3.34% referred to difficulties encountered during program implementation (Table 4), and 18.11% were recommendations (Table 5).

Regarding the conclusions related to the perceptions of the program, results in Table 1 showed that most of the responses were positive in nature in the areas of satisfaction, program content, program arrangement, and program implementation. For instance, *"Instructors appreciated the strong theoretical bases of the program"*, *"Students liked the games and activities in the lessons"*, and *"Every instructor agreed that the atmosphere in the lesson was very good"*. Among the 497 responses, 420 responses were classified as positive (84.51%). The intrarater agreement percentages on the positivity of coding were 100 and 95%, while the inter-rater agreement percentages on the positivity of coding were 95 and 95%. For the perceptions of the program implementers, findings in Table 2 also showed that most of the responses were positive in nature. For example, *"Both instructors and students perceived that the program implementers had good professional attitudes"*, *"Teachers were actively involved in the class activities"*, and *"Students perceived that instructors were caring"*. Among the 334 responses, 326 were positive (97.60%). The intrarater agreement percentages on the positivity of coding were 100 and 100%, while the inter-rater agreement percentages on the positivity of coding were 95 and 100%.

**TABLE 1**
**Responses Related to the Program Participants' and Instructors' Views on the Program**

| Category | Responses | Nature of the Response | | | | Total |
|---|---|---|---|---|---|---|
| | | Positive | Neutral | Negative | Undecided | |
| Satisfaction level | Satisfied with the program | 137 | | | | 137 |
| | Positive impression towards the program | 51 | | | | 51 |
| | Liked the program | 36 | | | | 36 |
| | Would suggest friends to join the program | 8 | | | | 8 |
| | Would join the program again | 4 | | | | 4 |
| | Satisfied with one's own performance in the program | 4 | | | | 4 |
| | Neutral comments | | 18 | | | 18 |
| | Negative comments | | | 15 | | 15 |
| | **Subtotal** | **240** | **18** | **15** | **0** | **273** |
| Program content | Clear objectives and strong theoretical support | 39 | | | | 39 |
| | Diversified teaching means | 33 | | | | 33 |
| | Comprehensive and systematic content | 24 | | | | 24 |
| | Interesting teaching materials/interactive activities | 20 | | | | 20 |
| | Content met students' needs and abilities | 8 | | | | 8 |
| | Attractive games | 5 | | | | 5 |
| | Up-to-date and detailed information | 5 | | | | 5 |
| | Other positive comments on content | 17 | | | | 17 |
| | Unable to match students' needs | | | 15 | | 15 |
| | Boring teaching format and materials | | | 9 | | 9 |
| | Unclear objectives and weak theoretical support | | | 4 | | 4 |
| | **Subtotal** | **151** | **0** | **28** | **0** | **179** |
| Program arrangement | Whole school cooperation | 4 | | | | 4 |
| | Time constraint | | | 10 | | 10 |
| | The sessions were too long/too many sessions | | | 4 | | 4 |
| | **Subtotal** | **4** | **0** | **14** | **0** | **18** |
| Program implementation | Good atmosphere | 14 | | | | 14 |
| | Sufficient discussion/reflection time | 6 | | | | 6 |
| | Had adequate support from the Project | 4 | | | | 4 |
| | Real cases sharing | 1 | | | | 1 |
| | Lack of discussion/reflection time | | | 2 | | 2 |
| | **Subtotal** | **25** | **0** | **2** | **0** | **27** |
| **Total responses** | | **420** | **18** | **59** | **0** | **497** |

**TABLE 2**
**Responses on the Views towards Program Implementers**

| Category | Responses | Nature of the Response | | | | Total |
|---|---|---|---|---|---|---|
| | | Positive | Neutral | Negative | Undecided | |
| Satisfaction level | Very satisfied | 10 | | | | 10 |
| | Satisfied | 110 | | | | 110 |
| | Very positive comments | 5 | | | | 5 |
| | Positive comments | 20 | | | | 20 |
| | **Subtotal** | **145** | **0** | **0** | **0** | **145** |
| Views about the instructors | Professional attitude | 9 | | | | 9 |
| | Commitment | 21 | | | | 21 |
| | Sufficient preparation/understanding of the program | 10 | | | | 10 |
| | Instructor's attitude and performance were influential to students' learning | 13 | | | | 13 |
| | Helpful | 12 | | | | 12 |
| | Caring | 10 | | | | 10 |
| | Had positive views towards students | 2 | | | | 2 |
| | Had positive views towards others | 4 | | | | 4 |
| | Satisfactory performance | 92 | | | | 92 |
| | Insufficient mastery of program/teaching skills | | | 8 | | 8 |
| | **Subtotal** | **173** | **0** | **8** | **0** | **181** |
| Others | Other positive comments on instructors | 8 | | | | 8 |
| | **Subtotal** | **8** | **0** | **0** | **0** | **8** |
| **Total responses** | | **326** | **0** | **8** | **0** | **334** |

Concerning the perceived effectiveness of the program to the students, as shown in Table 3, there were a total of 626 meaningful units that could be categorized in several categories, including societal, familial, interpersonal, and personal enhancement. These categories were developed according to the ecological perspective[37,38], which was adopted as one of the theoretical frameworks in the development of the Tier 1 Program. Examples of student development in the interpersonal and personal levels were *"Through this program, the relationships among the students could be enhanced"* and *"The Tier 1 Program could raise students' self-confidence specially"*, respectively. Overall, the positive effects of the program in different ecological domains were evident. Among 626 responses, 610 were positive (97.44%). The intrarater agreement percentages on the positivity of coding were 100 and 95%, while the inter-rater agreement percentages on the positivity of coding were 95 and 100%.

Despite the positive feedback, a small number of responses (n = 62) were related to difficulties encountered. For instance, *"Time is pressing in daily lessons. Even though students had interactions, they could not carry out in-depth discussion and investigation (on the topic)", "When having group activities, it was difficult to control classroom discipline", "The biggest difficulty the teachers encountered was how to strike a balance between maintaining original teaching materials and tailor-making in accordance with students' capabilities and emotional responses during implementation, so as to let students involve while not destroying the completeness of the program"*. All the responses were categorized into the aspects of program content, program format, time arrangement, and program implementation (see Table 4). The intrarater agreement percentages on the category of coding were 100 and 95%, while the inter-rater agreement percentages on the category of coding were 85 and 85%. Lastly, the suggestions for improvement can be seen in Table 5 (n = 336). It is noteworthy that some suggestions for improvement

**TABLE 3**
**Responses on Perceived Effectiveness**

| Category | Subcategory | Responses | Nature of the Response | | | | Total |
|---|---|---|---|---|---|---|---|
| | | | Positive | Neutral | Negative | Undecided | |
| Societal level | Social responsibility and identity | Enhanced students' social participation and sense of caring | 12 | | | | 12 |
| | | Enhanced national and racial identities | 1 | | | | 1 |
| | | **Subtotal** | **13** | **0** | **0** | **0** | **13** |
| Familial level | Family relationships | Enhanced students' relationship with their families | 3 | | | | 3 |
| | | **Subtotal** | **3** | **0** | **0** | **0** | **3** |
| Interpersonal level | General interpersonal competence | Improved interpersonal relationship | 69 | | | | 69 |
| | | Enhanced bonding with healthy adults | 2 | | | | 2 |
| | | Enhanced student-instructor relationship | 20 | | | | 20 |
| | | Enhanced peer relationship | 7 | | | | 7 |
| | | Increased communication between students | 12 | | | | 12 |
| | | Increased communication between instructors and students | 15 | | | | 15 |
| | | Enhanced understanding/mutual understanding | 9 | | | | 9 |
| | | **Subtotal** | **134** | **0** | **0** | **0** | **134** |
| | Specific interpersonal competence | Respect | 8 | | | | 8 |
| | | Communication skill | 7 | | | | 7 |
| | | Cooperation | 2 | | | | 2 |
| | | **Subtotal** | **17** | **0** | **0** | **0** | **17** |
| Personal level | Cherishing life | Treasuring of life | 2 | | | | 2 |
| | | Reflection of life | 17 | | | | 17 |
| | | **Subtotal** | **19** | **0** | **0** | **0** | **19** |
| | Cognitive competence | Promoted analytical ability | 5 | | | | 5 |
| | | Promoted cognitive ability | 2 | | | | 2 |
| | | **Subtotal** | **7** | **0** | **0** | **0** | **7** |
| | Positive self-image and development | Enhanced students' development | 190 | | | | 190 |
| | | Had some impacts on students' development | 11 | | | | 11 |
| | | Cultivation of resilience | 16 | | | | 16 |
| | | Mastery of future | 4 | | | | 4 |
| | | Goal setting | 1 | | | | 1 |
| | | Promoted self-efficacy | 3 | | | | 3 |
| | | Enhanced self-confidence | 7 | | | | 7 |
| | | Enhanced self-determination | 16 | | | | 16 |
| | | Enhanced self-understanding | 45 | | | | 45 |
| | | Enhanced sense of responsibility | 2 | | | | 2 |
| | | **Subtotal** | **295** | **0** | **0** | **0** | **295** |

*Table 3 continues*

**TABLE 3 (continued)**

| | | | | | | | |
|---|---|---|---|---|---|---|---|
| | Emotional competence | Promoted emotional control | 30 | | | | 30 |
| | | **Subtotal** | **30** | **0** | **0** | **0** | **30** |
| | Moral competence and virtues | Promoted ability of differentiating between right and wrong | 35 | | | | 35 |
| | | Strengthened positive values | 5 | | | | 5 |
| | | **Subtotal** | **40** | **0** | **0** | **0** | **40** |
| | Learning | Enhanced students' classroom participation | 16 | | | | 6 |
| | | Students gained extracurricular knowledge | 6 | | | | 3 |
| | | Promoted presentation ability | 3 | | | | 16 |
| | | **Subtotal** | **25** | **0** | **0** | **0** | **25** |
| Others | | Helpful to instructors | 16 | | | | 11 |
| | | Other positive impacts | 11 | | | | 16 |
| | | Neutral comments on program effectiveness | | 16 | | | 16 |
| | | **Subtotal** | **27** | **16** | **0** | **0** | **43** |
| **Total responses** | | | **610** | **16** | **0** | **0** | **626** |

**TABLE 4**
**Responses on Difficulties Encountered during Program Implementation**

| **Category** | **Responses** | **Total** |
|---|---|---|
| Program content | Too much content | 11 |
| | Could not match students' abilities/needs/interests | 9 |
| | Overlapping | 2 |
| | Not suitable to non-Chinese students' culture | 1 |
| | **Subtotal** | **23** |
| Program format | Negative comments on growth puzzle | 2 |
| | Too many written tasks | 2 |
| | **Subtotal** | **4** |
| Time arrangement | Time constraint | 14 |
| | **Subtotal** | **14** |
| Program implementation | Difficult to maintain students' discipline while teaching | 9 |
| | Unfavorable arrangement | 6 |
| | Spent much time for preparing the lessons | 4 |
| | High instructor-student ratio affected the program effectiveness | 2 |
| | **Subtotal** | **21** |
| **Total responses** | | **62** |

were contradictory (e.g., "deepen program content" vs. "simplify and condense the program content" under the category of program content). Simply based on the category of codes level, the intrarater agreement percentages were 90 and 90%, while the inter-rater agreement percentages were 80 and 85%.

**TABLE 5**
**Responses on Recommendations**

| Category | Descriptions | Total |
|---|---|---|
| Program content | Add interesting elements | 26 |
| | Adjust to the needs and abilities of students | 23 |
| | Improve content of teaching materials | 19 |
| | Deepen program content | 12 |
| | Simplify and condense the program content | 11 |
| | Be more applicable to real-life situations | 7 |
| | Conduct school-based program | 5 |
| | Improve topic coherence | 5 |
| | Match up with the social environment | 4 |
| | Need diversified content | 4 |
| | Provide revised supplementary materials | 4 |
| | Content should be more lively | 3 |
| | Improve the content of PowerPoint and worksheets | 3 |
| | Improve the linkage between program aims and program activities | 3 |
| | Need clearer objectives | 2 |
| | Content should be more attractive | 1 |
| | Provide English translation of the content | 1 |
| | **Subtotal** | **133** |
| Program format | Add more games/activities | 30 |
| | Add more multimedia teaching aids | 17 |
| | Need more diversified format | 12 |
| | Increase flexibility | 11 |
| | Add more interactive format | 9 |
| | Flexible discussion topics/prolong discussion and sharing time | 5 |
| | Improve growth puzzle | 5 |
| | Decrease the quantity of worksheets | 4 |
| | Add students' self-reflection and sharing | 4 |
| | Improve the arrangement | 3 |
| | Add more stories | 1 |
| | Set up reward and penalty system | 1 |
| | **Subtotal** | **102** |
| Time arrangement | Match up content and time | 28 |
| | Regulate activity time | 16 |
| | Prolong duration of lesson | 10 |
| | **Subtotal** | **54** |
| Implementation | Provide more training and assistance to instructors | 5 |
| | Strengthen follow-up and consolidation work | 5 |
| | Consolidate teaching experience systematically | 4 |
| | Enhance the collaboration between teachers and social workers | 3 |
| | Adjust instructor-student ratio | 3 |
| | Decrease intensity and number of lessons | 2 |
| | Provide more support to schools | 2 |
| | Enhance classroom discipline/environment | 1 |
| | Incorporate into the formal curriculum | 1 |
| | Improve the review form | 1 |
| | **Subtotal** | **27** |
| Others | Other recommendations | 20 |
| | **Subtotal** | **20** |
| **Total responses** | | **336** |

### DISCUSSION

Utilizing secondary data analyses, this study attempted to analyze the conclusions drawn by the program implementers of the Tier 1 Program of the Project P.A.T.H.S. in the first year of the Full Implementation Phase. Several findings were observed. First, in line with previous findings of secondary data analyses[10], results showed that the majority of the responses related to the perceptions of the Tier 1 Program (Secondary 1 Curriculum), instructors, and program effectiveness were positive in nature. These findings, based on secondary data analyses of subjective outcome evaluation, are consistent with those of previous evaluation studies on the Project P.A.T.H.S. using different evaluation mechanisms. For instance, both quantitative longitudinal objective outcome evaluation[4,5] and qualitative analyses of students' weekly diaries[15] showed that the program participants identified the benefits of the programs to their psychosocial development. Also, qualitative case studies[16,17] and interim evaluation[20,21] showed that the instructors perceived the program and themselves as program implementers positively, although difficulties encountered during implementation were also noted. In addition, the present findings, based on a larger sample size than the previous study[10], appear to be more representative in demonstrating the merits and benefits of the Project P.A.T.H.S. in Hong Kong. Consistent with the spirit of the utilization-focused approach[24], the views of the program implementers were taken into account as well as the reports that they prepared. Since similar evaluation studies were commonly conducted in the West[25,26,27], the present study was an addition to the existing literature, particularly in the Chinese culture.

Apart from this, the present findings revealed that program implementers had come across several difficulties in program execution and had suggested corresponding recommendations for improvement in the reports. These additional findings were expected because secondary data analyses allow researchers to "present interpretations, conclusions, or knowledge that are additional to or different from those presented in the first report on the original inquiry as a whole" (p. 260)[39]. In fact, difficulties and problems encountered during program implementation were anticipated because the project was launched in the first year of the Full Implementation Phase, and only 52 of the 207 participating schools had gained prior program implementation experience through joining the Experimental Implementation Phase. Similar to those findings in the first year of the Experimental Implementation Phase[10], the difficulties reported were generally related to program content, program format, time arrangement, and program implementation.

As the Project P.A.T.H.S. is a pioneering, positive youth development program using a curricular-based approach, it is understandable that fitting the program into the school teaching timetable and completing the activities within time limits would be a challenge. Moreover, program implementers need to spend time preparing such a new curriculum, and sometimes have to tailor-make the program to match their students' needs and interests whenever necessary. Furthermore, in such a kind of experiential learning, both teachers and social workers need to adapt to new teaching methods to let students experience and learn. For instance, apart from one-way instruction, they have to facilitate student discussion, perform role play, and even disclose themselves as a kind of story telling. They need to adopt a flexible approach to carry out structured activities in the classrooms, while maintaining classroom discipline. As such, with reference to the difficulties encountered, recommendations on program content (e.g., adding interesting elements and adjusting the content to meet students' needs), program format (e.g., adding more games and activities), time arrangement (e.g., matching up content with time), and implementation (providing more training and assistance to instructors) were observed. Constructively speaking, the problems encountered and recommendations for improvement can serve as useful pointers to fine tune the program to cater to the needs of program implementers and participants.

Although the present findings can be interpreted as evidence supporting the effectiveness of the Project P.A.T.H.S., several alternative explanations are present. The first alternative explanation is that the findings are due to insufficient evaluation expertise of the program implementers. However, this can be dismissed because social workers and teachers had learned about program evaluation in their professional training, and for those who joined the Project P.A.T.H.S., they had received specific training in data collection and report writing prior to program implementation and evaluation. In addition, evaluation manuals and report templates were provided to all participating schools as self-help tools, which helped program implementers to familiarize themselves with the steps of data collection, analyses, and report writing. Moreover, the program implementers could call the research team for advice when encountering difficulties. Therefore, it is believed that the program implementers were able to integrate the subjective outcome evaluation findings and translate them into meaningful conclusions, and thus credibility of the data collection and reports was high. The second alternative explanation is that the findings are due to biases (e.g., cognitive dissonance, rice bowl, and revenge arguments). However, an examination of the present findings showed that the conclusions drawn by the program implementers were in line with other evaluation findings reported by the research team. Since an integration of different existing findings has painted a consistent picture of the program effectiveness based on the principle of triangulation, it indicates that the influences of biases are minimal.

There are several strengths of using qualitative secondary data analysis[31,32,33,40]. First of all, it is economical in terms of money, time, and effort of data collection. Also, the database is large and readily accessible. Moreover, the information regarding the data collection process is available and documented. In addition, the data collected by open-ended questions are not limited by the preset question-and-answer format of the survey data, and are not affected by the possible investigator's biases appearing in the interviews.

However, several criticisms could be leveled against studies that utilize secondary data analyses such as the present study[31,32,33,40]. First, since secondary data analyses utilize an existing database, it is not possible to have interactive collaboration with the program implementers to explore further related issues. Second, the conclusions written were brief, and could hardly provide an in-depth understanding of the implementation process or school administration. Therefore, it is valuable to conduct school-based case studies to document exemplary school administration and program implementation. Third, although the five conclusions generated from each evaluation report gave an overall picture of each school, they could not give detailed descriptions of the perceptions of individual program implementers and participants. Thus, it is more illuminating to carry out in-depth interviews with instructors and students. Despite these limitations, the existing research findings suggest that both program participants and implementers had positive perceptions of the program and regarded the program as helpful to the students' overall development. In short, merits and benefits associated with the Project P.A.T.H.S. Tier 1 Program are evident.

### ACKNOWLEDGMENTS

The preparation for this paper and the Project P.A.T.H.S. were financially supported by The Hong Kong Jockey Club Charities Trust.
